# Geochemical fingerprinting of Pleistocene stone tools from the Tràng An Landscape Complex, Ninh Bình Province, Vietnam

**DOI:** 10.1371/journal.pone.0269658

**Published:** 2022-06-22

**Authors:** Benjamin Utting

**Affiliations:** Department of Archaeology, University of Cambridge, Cambridge, United Kingdom; Sapienza University of Rome: Universita degli Studi di Roma La Sapienza, ITALY

## Abstract

Raw material analyses of prehistoric stone tool assemblages can reveal insight into mobility and exchange patterns in hunter-gatherer populations by reconstructing the circulation of stone throughout ancient landscapes. In Pleistocene Southeast Asia, stone tools are generally thought to have been fashioned from easily accessible local raw materials. However, despite the consistent presence of stone tools made of igneous raw material at prehistoric sites throughout the Tràng An Landscape Complex in northern Vietnam, there are no sources of igneous raw material in the immediate vicinity. This paper presents the results of geochemical sourcing analysis of late Pleistocene igneous stone tools from Tràng An: the first analysis of its type in mainland Southeast Asia. The results shed light on mobility and raw material provisioning strategies in Pleistocene mainland Southeast Asian hunter-gatherer populations and raise questions surrounding the relationship between technological organization, raw material, and expediency in Southeast Asian stone tool assemblages.

## Introduction

Late Pleistocene stone tool assemblages recovered from sites in mainland Southeast Asia have a history of being discussed in the context of a prevailing archaeological technocomplex called the ‘Hoabinhian’ [1–6]. While these assemblages usually contain a proportion of tools that can be and have been assigned to a formal typology (e.g., sumatralith, short axe), they consist primarily of expediently reduced stone tools exhibiting limited evidence of retouch or consistent morphological design. Historical characterizations of both components of Hoabinhian stone tool assemblages have generally portrayed them as simplistic and crude, giving rise to early interpretations that they represented evidence for cognitive or cultural stagnation [1, 7, 8]. This paradigm is now considered to be inaccurate. Among recent works, the applications of use-wear analysis, residue analysis, and geometric morphometric approaches have revealed that a significant degree of complexity and behavioral adaptability is accessible through these assemblages and Southeast Asian stone tools more broadly [9–12]. However there continues to be little discussion about raw material variability, as stone tools have been generally assumed to be fashioned from locally provenanced material [3, 13–15]. Assemblages from sites investigated in the Tràng An Landscape Complex include the same characteristically low incidence of forms that fall into traditional Hoabinhian formal tool types and an overwhelming majority that appear expedient [16, 17]. During the course of investigation by the author it was found that a proportion of pieces, both formal and informal, were made on rounded igneous cobbles. With no local source of igneous raw material or a mechanical means of cobble formation [18], this presented an ideal case study to explore raw material sourcing and mobility more closely. There were three analytical objectives to the current study. The first objective was to geochemically classify lithics from Tràng An. While some lithics have been previously issued with petrous classifications (e.g., basalt, rhyolite, diabase), it is difficult to assess the validity of these groupings without rigorous methods of identification. The second objective was to identify possible geological sources of the raw material, and the third: to evaluate the potential of portable X-Ray Fluorescence Spectroscopy (pXRF) as an approach to assessing raw material (and by extension behavioral) variability between and within assemblages that might ordinarily be subsumed under a “Hoabinhian” classification.

### Investigating mobility

The analytical objectives of the paper are underpinned by theoretical considerations regarding our understanding of prehistoric tropical forager mobility. One of the primary goals of this research has been to gain insight into the mobility strategies of ancient hunter-gatherers at Tràng An and their potential wider implications. Two broad types of prehistoric hunter-gatherer mobility strategies have been prevalent in the literature since the 1980’s. In this brief opening review I explore archaeological correlates associated with each of them [19–23]. The first, termed “residential mobility”, is characterized by the movement of entire groups to different areas; the second, termed “logistical mobility”, sees small subgroups radiate away from a large central camp to smaller satellite camps for short-term stays to collect resources to bring back to the base camp. Archaeological expectations of residential mobility strategies include expedient tools made of generally locally available raw material (place provisioning). Conversely, archaeological expectations accompanying logistical mobility strategies include tools made of non-local raw materials (self-provisioning) that have been more efficiently reduced [19, 24, 25]. Differences in hunter-gatherer mobility strategy can be attributed to several factors, including shifts in local environment, changes in demography, or the transition from foraging to farming [21]. Most of the research into hunter-gatherer mobility strategies has focused on temperate to arctic zones. Furthermore, 20^th^ century research suggested that rainforests were generally unsuitable for human occupation without the aid of agriculture [26, 27]. However, a growing body of evidence demonstrates that tropical rainforests were key environments in human evolution, and that humans have actively altered rainforest environments for at least 45,000 years [28–32]. Therefore, a greater emphasis on understanding mobility in tropical hunter-gatherer populations has the potential to significantly augment our understanding of the role of tropical environments in human evolution and dispersal.

In seasonal tropical mainland Southeast Asia, Shoocongdej [33] suggests that, due to dramatic environmental changes associated with the seasonal monsoon, ancient mainland Southeast Asian hunter gatherers practiced different types of mobility at different times of the year: residential mobility during the wet season and logistic mobility during the dry season. Shoocongdej proposes several archaeological correlates for each mobility system. For the first (wet season, residential mobility), archaeological expectations include a) higher floral and faunal resource diversity, b) unbiased representation of faunal remains, c) small and multifunctional toolkits, d) limited use of storage technologies, and e) predominantly local lithic raw materials and expedient stone reduction methods. For the second, archaeological expectations include a) lower diversity of floral and faunal remains, b) strongly biased representation of faunal remains (i.e., body parts), c) highly specialized toolkits, d) low general diversity of artifacts/floral/faunal remains, e) presence of storage and caching facilities, and f) non-local lithic raw materials and curated stone technology.

Assemblage-wide investigations into tropical forager mobility in mainland Southeast Asia remain a rarity. With few exceptions, such as Marwick’s 2013 study tracking the relationship between technological variation and environmental change to test the validity of behavioral ecological models of forager behavior at Tham Lod and Ban Rai rockshelters in Thailand, the majority of studies [14, 15, 34] tend to be more acutely focused on technological characterizations and diagnosing the *chaîne opératoire* for each assemblage. The application of pXRF represents a novel approach to the study of raw material sourcing and the still poorly understood nature of tropical forager mobility in Southeast Asia.

## Research setting

**Fig 1:**
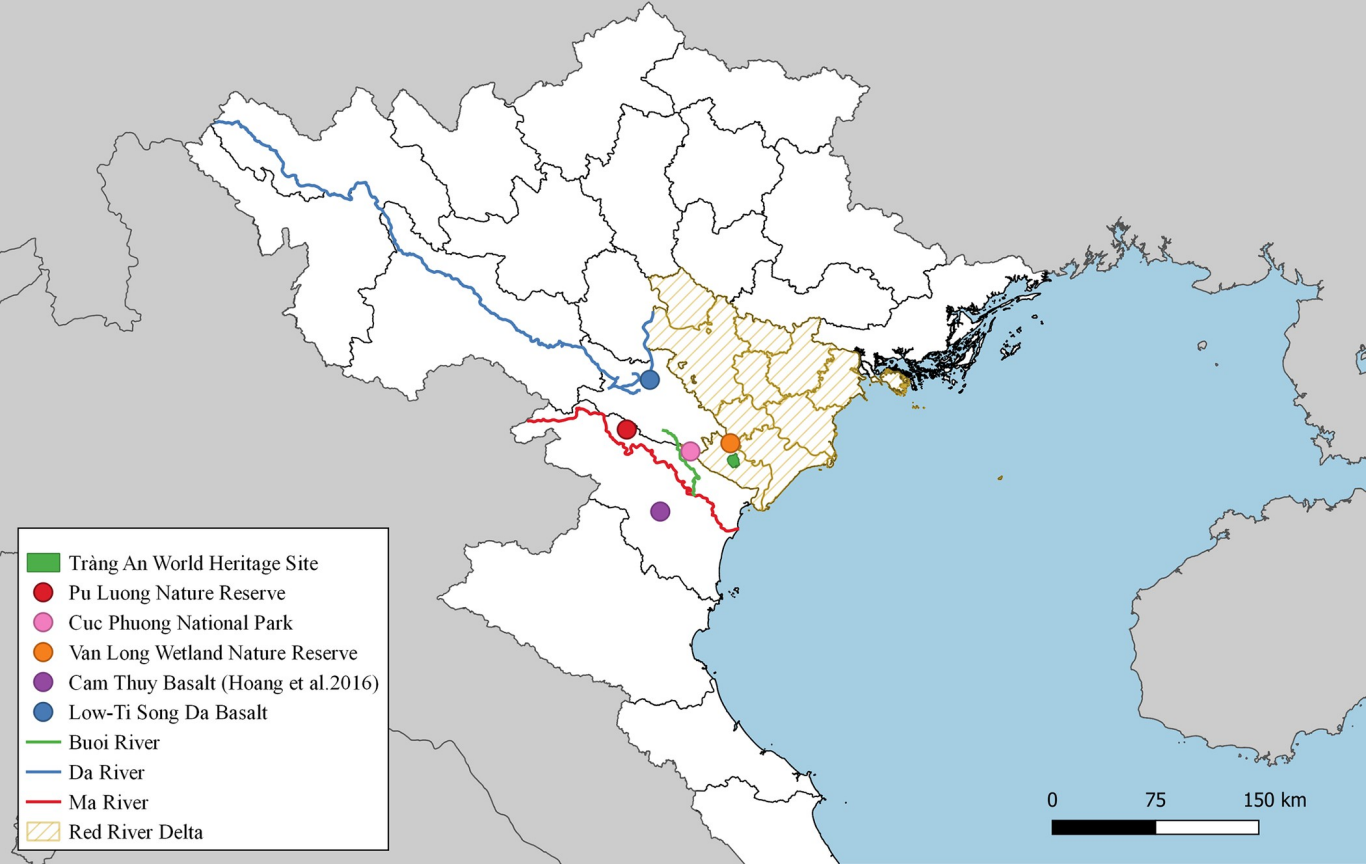
Map of northern Vietnam with key sites mentioned in this paper. Shapefiles reprinted from the Database of Global Administrative Areas and the Stanford Digital Repository under a CC BY license, with permission from the Database of Global Administrative Areas (http://www.gadm.org) and the Stanford Digital Repository (http://www.sdr.stanford.edu). The figure was made with QGIS 3.20.1 under a CC BY license, with permission from QGIS (http://www.qgis.org).

**Fig 2:**
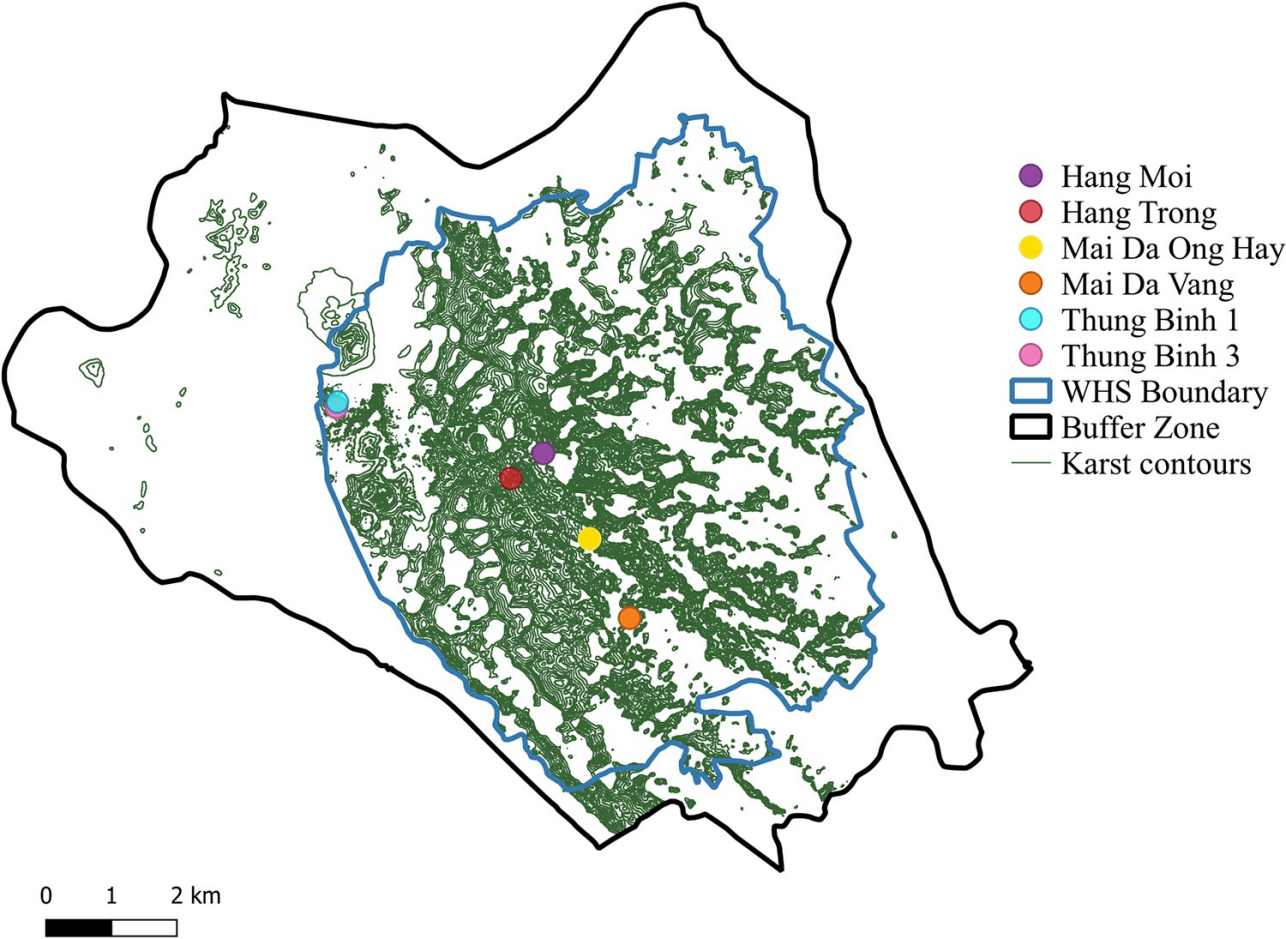
Contour map of Tràng An with excavated archaeological sites. “WHS” refers to World Heritage Site. Shapefile reprinted from GIS data obtained by the SUNDASIA Project, reprinted under a CC BY license with permission from the SUNDASIA Project. The figure was made with QGIS 3.20.1 under a CC BY license, with permission from QGIS (http://www.qgis.org).

The Tràng An World Heritage Complex is located at the southern margin of the Red River (Song Hong) Delta, approximately 85 km south of Hanoi in Ninh Bình Province, Vietnam (Figs 1 and 2). The property covers 6,226 hectares of Triassic limestone karstic landscape and is surrounded by a buffer zone of 6,026 hectares [35]. Archaeological investigations at cave and rockshelter sites throughout Tràng An have revealed consistent occupation of the complex for at least 37,000 years [36]. Palaeoenvironmental reconstruction [17, 37–40] suggests that the karstic ecology of Tràng An remained relatively resistant to large scale environmental instability associated with coastal inundation and global climate change, making it a potential refugial area for prehistoric populations. This means that Tràng An may have served as a key area where stable resource distribution encouraged long term, consistent occupation.

Previous analyses of lithic assemblages from Tràng An [16–18, 41–46] have revealed intra-assemblage raw material variability. While the majority of stone tools from Tràng An are made of locally sourced siliceous limestone, there are small but significant proportions of lithics made of non-local raw material (igneous/metamorphic stone, sandstone) at every site studied by the author (Table 1). Local igneous/metamorphic stone is a common raw material in Palaeolithic Southeast Asian stone tool assemblages [3, 13]; however, there are no known sources of igneous raw material within the immediate vicinity of Tràng An.

**Table 1:** Raw material proportions at sites throughout Tràng An. Percentage composition in parentheses.

Site Name and Total Lithics	Limestone lithics	Igneous lithics	Quartz lithics	Sandstone lithics
Hang Trong (N = 444)	339 *(76*.*4%)*	82 *(18*.*5%)*	6 *(1*.*3%)*	17 *(3*.*8%)*
Hang Thung Binh 1 (N = 604)	468 *(77*.*5%)*	109 *(18%)*	25 *(4*.*1%)*	2 *(<1%)*
Hang Thung Binh 3 (N = 264)	152 *(57*.*6%)*	104 *(39*.*4%)*	1 *(<1%)*	7 *(2*.*7%)*
Hang Moi (N = 99)	81 *(81*.*8%)*	15 *(15*.*2%)*	2 *(2%)*	1 *(1%)*
Mai Da Vang (N = 436)	428 *(98*.*2%)*	7 *(1*.*6%)*	0 *(0%)*	1 *(<1%)*
Mai Da Ong Hay (N = 335)	294 *(87*.*8%)*	33 *(9*.*9%)*	5 *(1*.*5%)*	3 *(<1%)*

**Fig 3:**
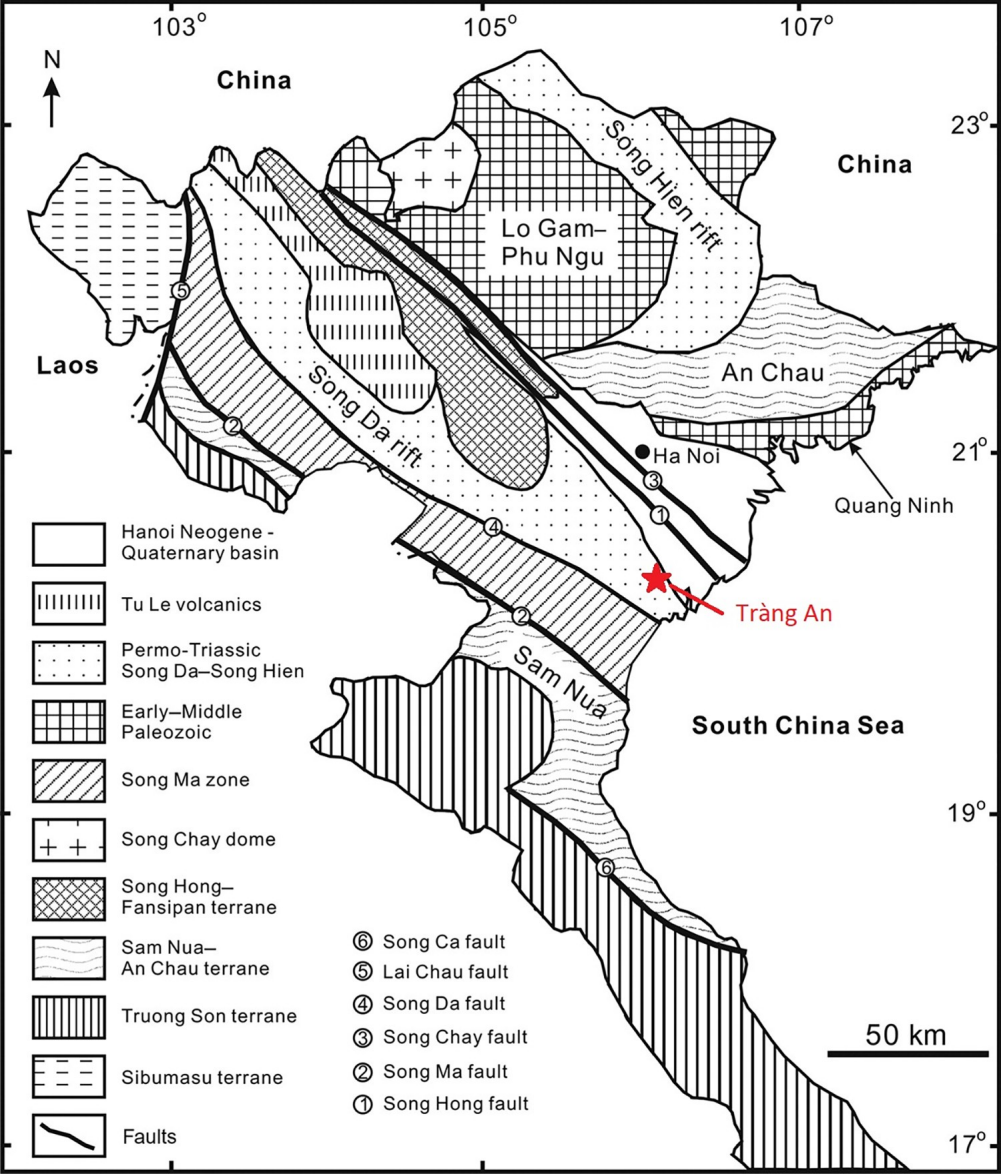
Geological map of Vietnam with Tràng An highlighted modified after Hieu et al. 2012 [55] under a CC BY license, with permission from Taylor & Francis Ltd, (http://www.tandfonline.com), original copyright 2012.

In order to investigate the source of this raw material, potential locales would need to fulfill two major criteria. The first is the presence of igneous stone. There are only two geological zones that are likely to have been a primary geological source in this case, as they encompass the only two igneous deposits that are accessible in the Red River Delta. The first is the Song Ma zone and the second is the Song Da rift structure (Fig 3). The second criterion is an environmental situation in which raw material would be worn down into round cobbles, as igneous stone tools from Tràng An have cortex that is consistent with fluvial depositional conditions. There are many rivers and streams associated with both geological structures, so it is likely that cobbles of appropriate size could be readily acquired either from primary or secondary depositions.

Geological studies of the southwestern Red River Delta divide igneous stone found there into two main categories based on geological formation. The Song Ma zone, created by the collision of the South China and Indochina blocks during the Indosinian orogeny in the middle Triassic [47, 48] and the Song Da rift structure, which is related to Permian volcanism associated with the formation of the Emeishan large igneous province [49–53]. Igneous stone from the Song Ma zone includes ophiolites (gabbro, basalt and metabasalt) that bear geochemical similarity to mid-ocean ridge basalt [54]. Igneous stone from the Song Da rift structure is broadly divided into two categories: low-Ti basalt (TiO_2_ wt% < 1.0) and high-Ti basalt (TiO_2_ wt% > 1.0). High-Ti basalt is considered to originate from enriched mantle sources whereas low-Ti basalts are thought to originate from depleted mantle sources [53].

The closest known source of igneous cobbles to Tràng An is from the Cam Thuy formation, accessible as close as 35 km southwest of Tràng An along the banks of the Ma River [56, 57]. The Cam Thuy formation is part of the Song Da rift structure but is only composed of high-Ti (TiO_2_ wt% > 1.0) basalt. Other candidate formations include the Song Ma formation (part of the Song Ma zone), accessible as close as 50 km west of Tràng An, and other outcrops of the Song Da rift structure that include low-Ti basalt (Fig 1). Dike structures emanating from primary geological formations may extend the range a few kilometers closer to Tràng An, but a foot survey of the western region of Cúc Phương National Park (~35 km northwest of Tràng An) and a boat survey of the southwestern region of the Van Long Nature Reserve (~12 km north) in 2018 and 2019 (respectively) by the author both failed to produce any evidence of igneous stone. Previous geological studies of Cúc Phương also note the predominance of carbonate rock and the absence of any igneous stone [56, 58]. Though relatively close to Tràng An, the Red River was not mentioned as a potential source of igneous cobbles in discussions with colleagues from the Vietnam Institute of Geosciences and Mineral Resources (pers. comm., Tran Tan Van & Nguyen Dai Trung), nor is it mentioned as a possible source in published literature.

A 2009 archaeological study of Palaeolithic stone tools from Con Moong Cave in Cúc Phương National Park examined igneous raw material variability through thin section analysis [59]. The results demonstrated the exploitation of a wide range of igneous river cobbles, including andesite, basalt, and obsidian. The authors hypothesize that the cobbles come from the Buoi River, which runs along the southwestern margin of the park, but do not consider the geological formation that the cobbles originate from. Parts of the Cam Thuy formation are the closest outcrops to Cúc Phương, and the Ma River lies slightly further afield from the Buoi River. Therefore, the likeliest candidate for this raw material is igneous stone from the Cam Thuy formation.

Based on proximity and previous studies, it was hypothesized that the stone tools made of rounded igneous cobble recovered from archaeological sites at Tràng An could be geochemically attributed to basalt from the Cam Thuy formation. Geochemical data are available for southwestern Cam Thuy basalts [52], so the decision was made to assess a previously unsampled source of Cam Thuy basalt in the Pù Luông Nature Reserve, roughly 80 km northwest of Tràng An to test this hypothesis.

## Materials

**Fig 4:**
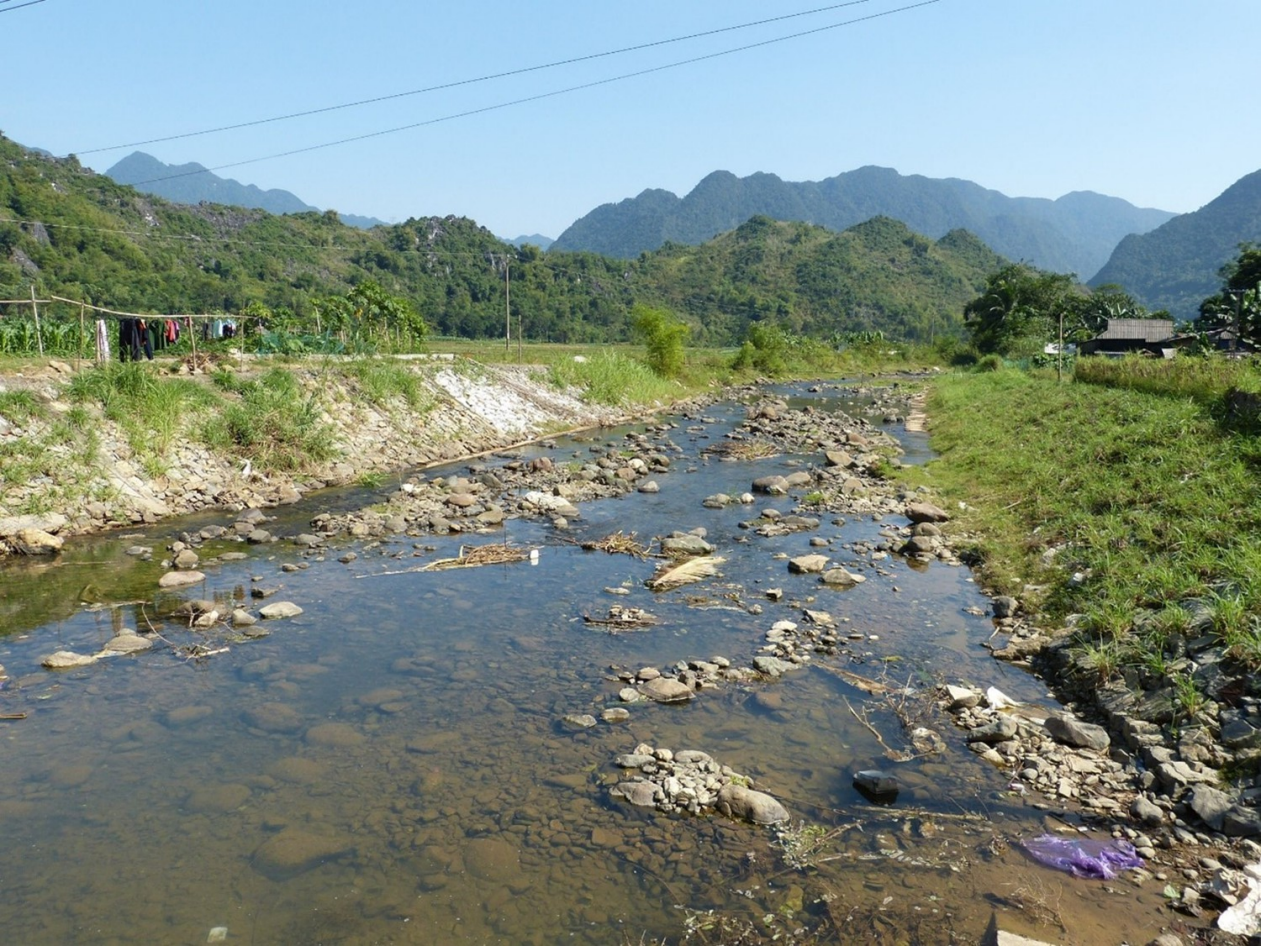
Sampling Location 1, Pù Luông Nature Reserve (Photo: Ryan Rabett).

**Fig 5:**
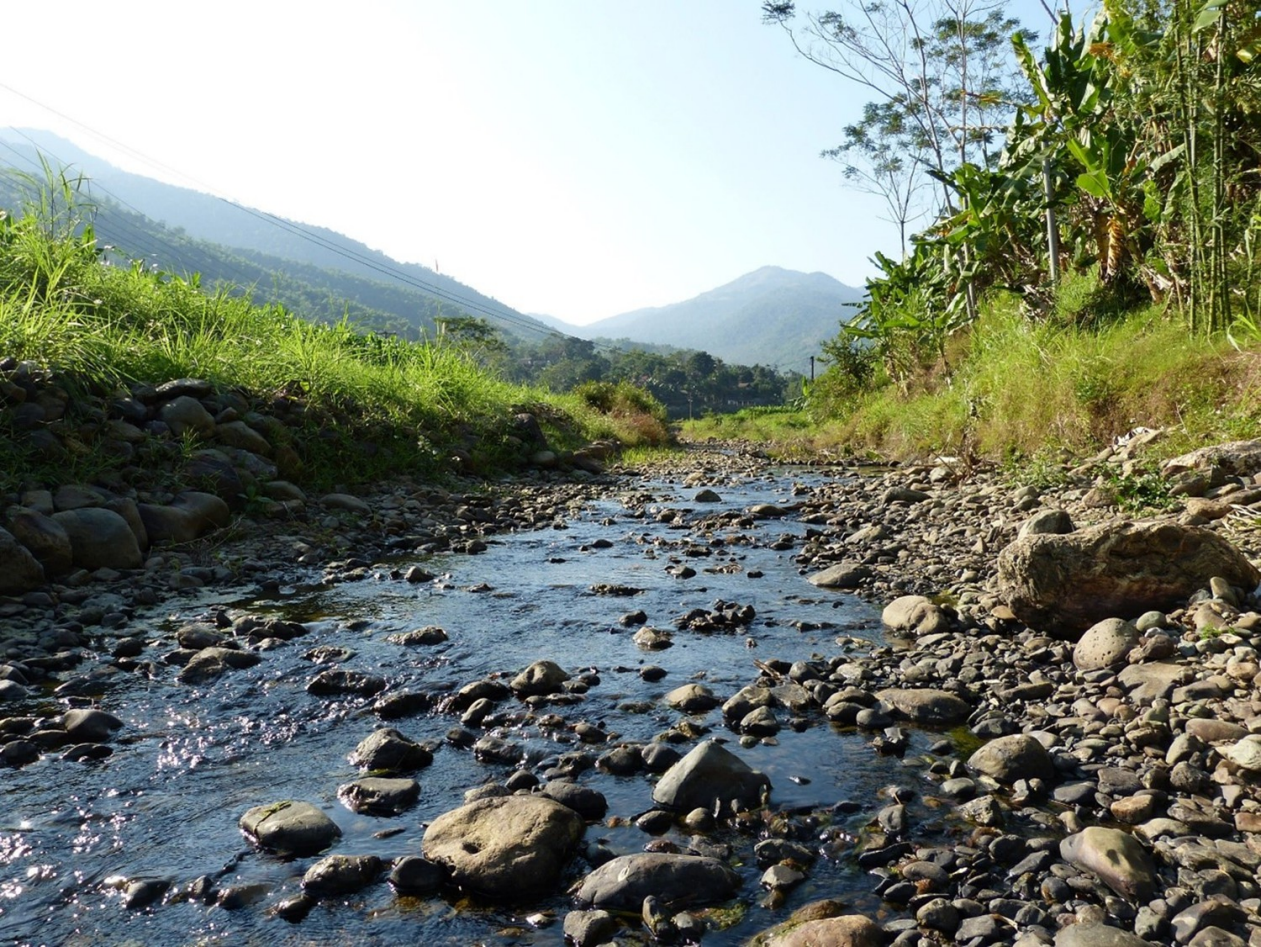
Sampling Location 2, Pù Luông Nature Reserve (Photo: Ryan Rabett).

**Fig 6:**
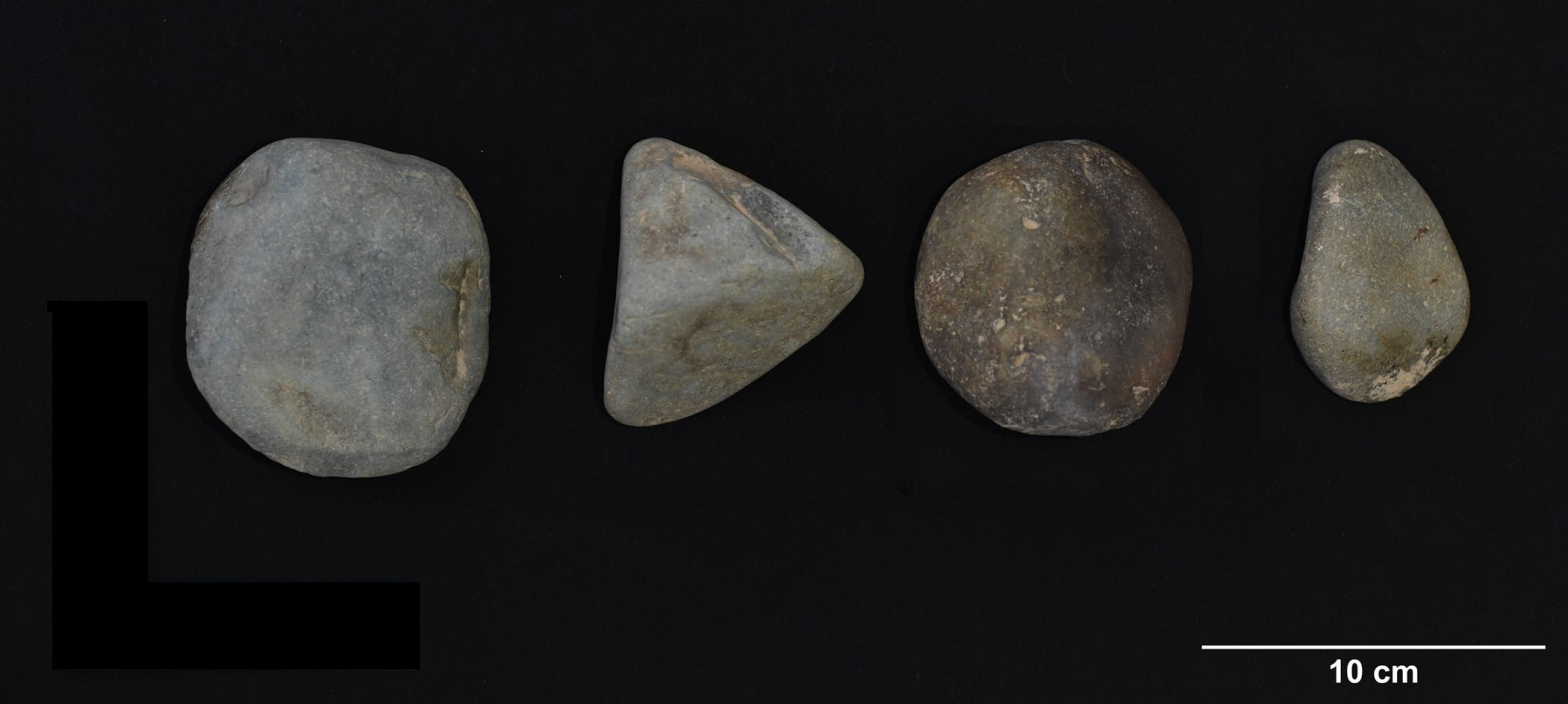
Basalt cobbles recovered from sampling location 1, Pù Luông Nature Reserve (photo by author).

**Fig 7:**
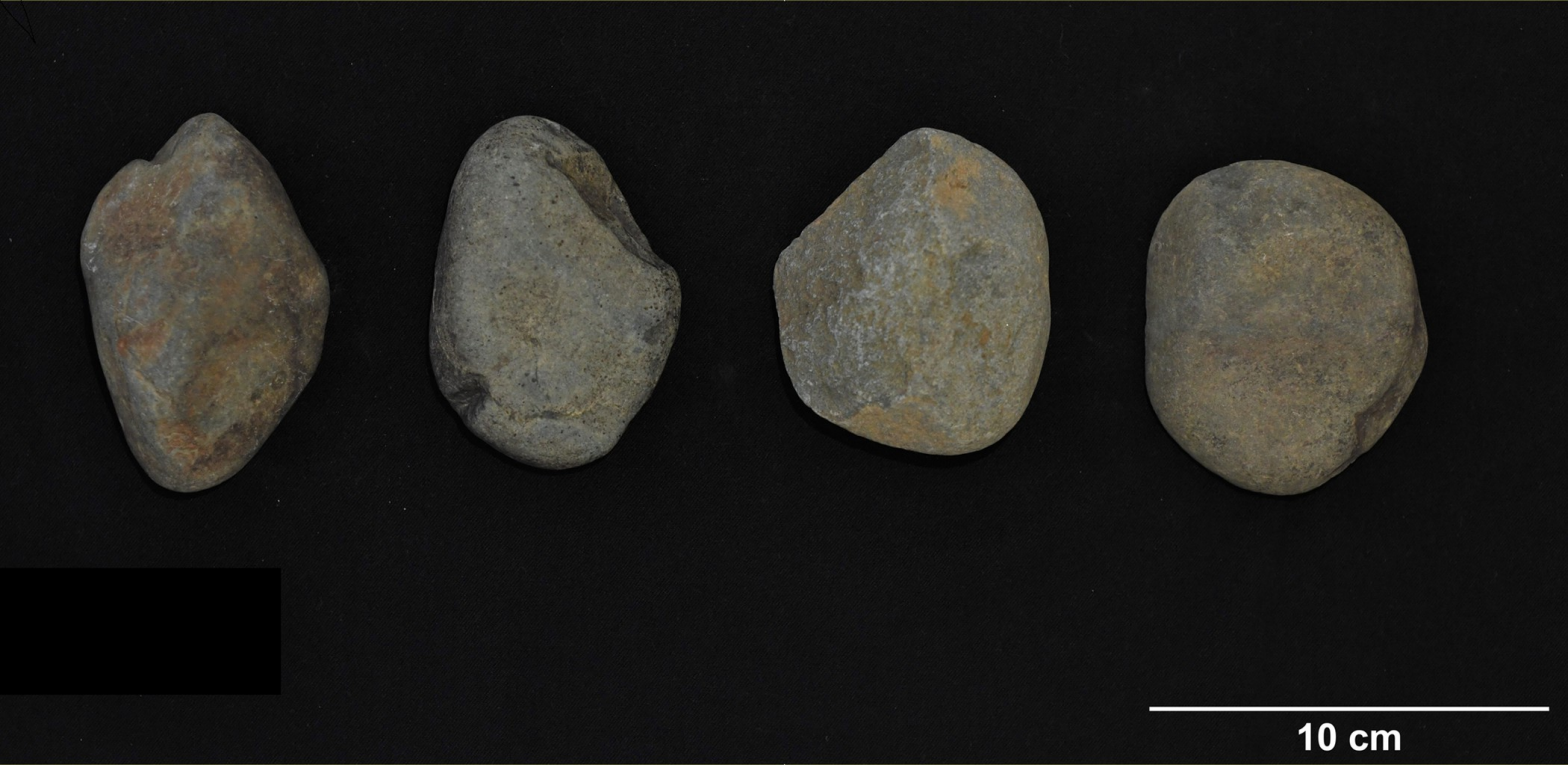
Basalt cobbles recovered from sampling location 2, Pù Luông Nature Reserve (photo by author).

In November 2019, a survey was conducted to collect raw material samples from the Pù Luông Nature Reserve. Two sampling locales were chosen within the reserve (L1: 20.452389N, 105.173139E & L2: 20.411294N, 105.188667E) (Figs 4 and 5). Both locales are small riverbeds with large numbers of basalt cobbles bearing visual resemblance to archaeological material from Tràng An. An initial sample of four cobbles was taken from L1 (Fig 6), with a larger sample of twenty-seven cobbles retrieved from the near-by and more heavily populated stream bed at L2 (Fig 7). Two survey methods were employed. The first was to walk along the riverbank and randomly select cobbles of similar color and cortical texture to that seen on stone tools from Tràng An. The second method, only exercised at L2, was to isolate a square meter and retrieve the surface scatter within the sampling area. The raw material samples range in mass from 25.5 g to 1,469.6 g and in length from 45.32 mm to 150.34 mm. The imbalance in sample sizes between each locale can be attributed to accessibility of stone along each riverbank. No permits were required for required for the described study, which complied with all relevant regulations.

The Tràng An excavated material selected for geochemical analysis comprised 33 stone tools from Hang Trong (HTC), 87 from Hang Thung Binh 1 (TB1), and 37 from Hang Thung Binh 3 (TB3). These sites were selected because they contain the highest proportion of igneous stone tools and cover a wide range of dates. Radiocarbon dates presented in this section were calibrated with IntCal20 through the CALIB 8.20 program [60] unless otherwise noted.

Archaeological material analyzed in this study is currently being stored between facilities at the Tràng An Visitor’s Center, Ninh Binh province, Vietnam and at the University of Cambridge, United Kingdom on authority of an agreement between the SUNDASIA Project, the Ninh Binh Provincial People’s Committee and the Tràng An Management Board. Each specimen has a unique identifier that is available in the data supplement (S1 File). All archaeological material will be returned to the Tràng An Visitor’s Center/Museum at the Tràng An Landscape Complex upon completion of analysis. All necessary permits for analysis of archaeological materials were obtained for the described study, which complied with all relevant regulations.

**Fig 8:**
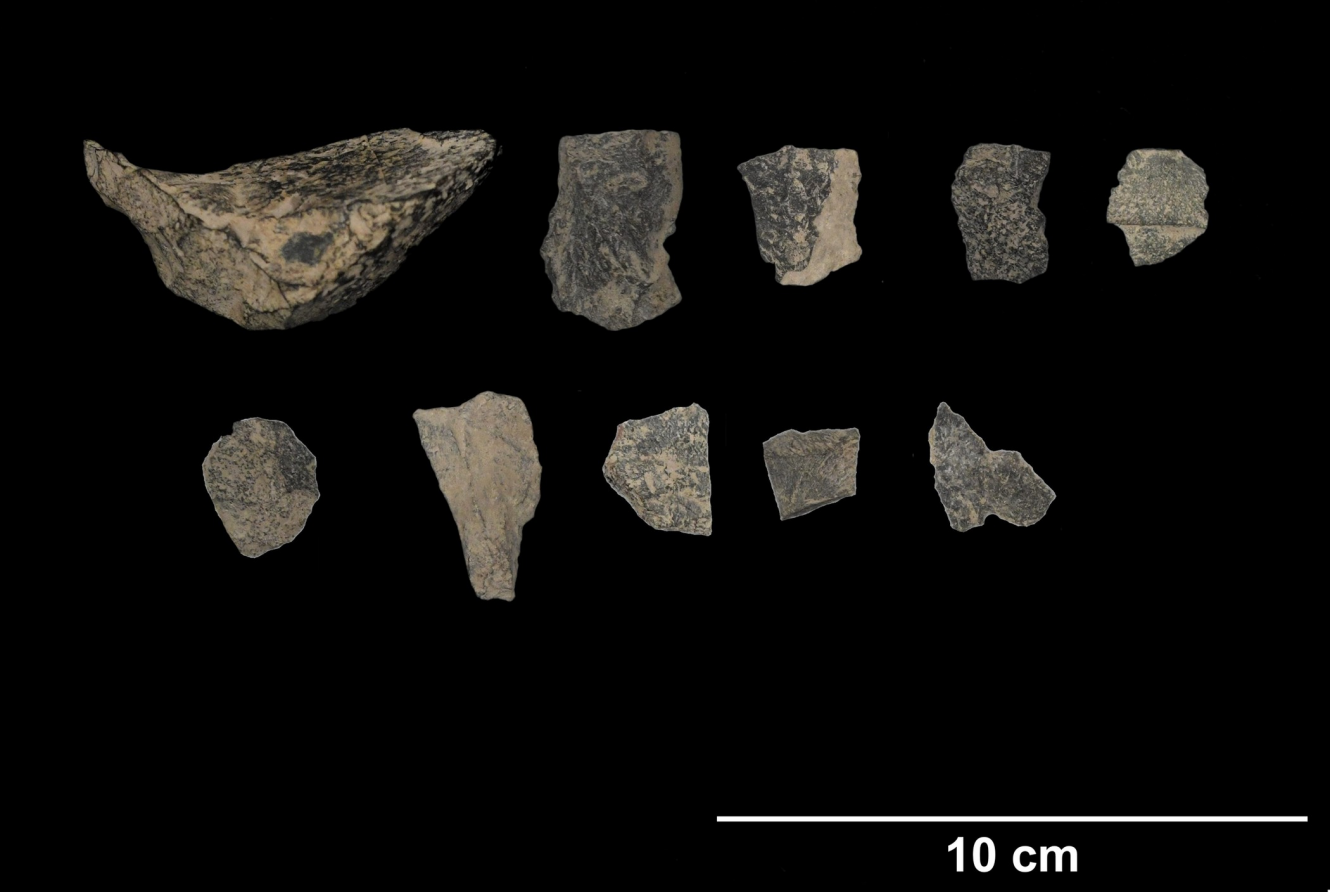
Lithics from Hang Trong (clockwise from upper left): Core tool, flake tools (photo by author).

HTC is located in the central area of the Tràng An massif (20.250444˚N, 105.890111˚E) and lies approximately 142 meters above sea level. It was first excavated in 2009–2010 and 2014 by the Tràng An Archaeological Project. There are three excavated trenches at HTC, and material from this study includes 32 specimens from trench 1 (center of the cave) and 1 from trench 3 (eastern wall of cave). Specimens from trench 1 were excavated from contexts radiocarbon dated to between 37,676–18,115 cal. BP (lab codes UBA-35063 and UBA-14884). The specimen from trench 3 was excavated from a cyclophorid shell midden assumed to be contiguous with a *Cyclophorus* shell midden context in trench 1, dated to 18,757–15,736 cal. BP (lab codes UBA-14886 and UBA-14884). Previous analyses of archaeological material from HTC suggests that it might have been used as a temporary stopping point or as a corridor between valleys in the complex [17]. The abundance of cyclophorid shells might suggest that the site was primarily occupied during the wet season. Other zooarchaeological evidence from HTC includes Cervidae, Cercopithecidae, Mustelidae, Hystricidae, Sciuridae, Manidae, Phasianidae, Geoemydidae, and unidentified fish remains. Igneous stone tools from HTC (Fig 8) are dark brown to black, fine grained, and have highly naturally polished cortex. There are no visible inclusions. Specimens included in this study include 13 complete flakes, 4 cores, 8 flake fragments, and 8 pieces of shatter.

**Fig 9:**
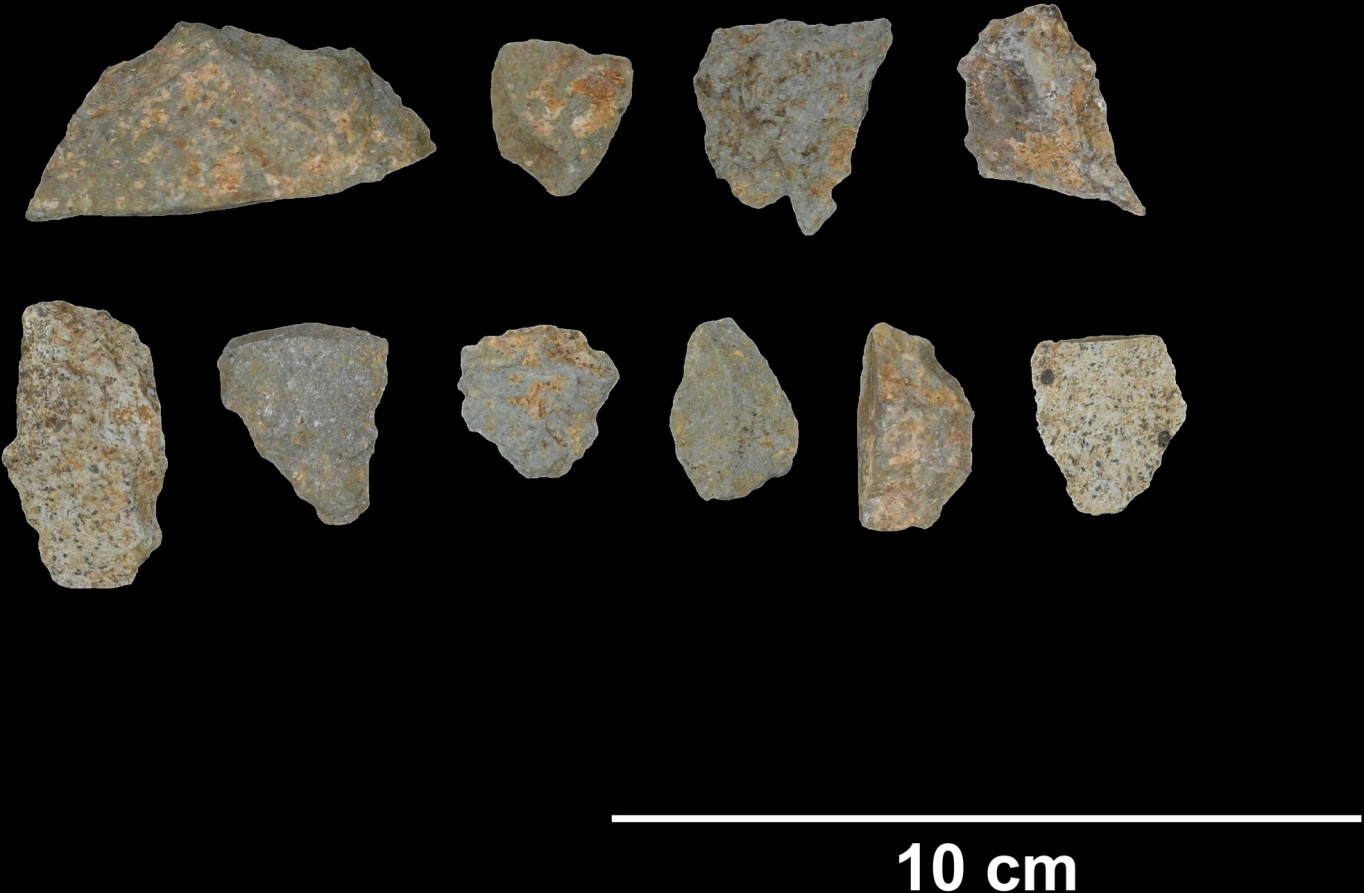
Lithics from Thung Binh 1 (clockwise from upper left): Short axe, flake tools (photo by author).

TB1 (20.26162N, 105.86474E) is situated in an isolated karstic formation in the northwest corner of the complex, approximately 1 km west of the main massif and c. 27 meters above sea level. It was first excavated by the Vietnam Institute of Archaeology in 2012 and subsequently by the SUNDASIA Project from 2017–19 [39]. Material analyzed in this study comes from a trench excavated towards the mouth of the main chamber. Samples in this study were excavated from a *Cyclophorus*-dominated shell midden dated to between 18,634–12,739 cal. BP (lab codes UBA-40554 and UB-38671). Notable faunal evidence from TB1 includes Cervidae, Hystricidae, Panthera, and Geoemydidae [39, 61]. The abundance of *Cyclophorus* shells paired with faunal evidence suggests that TB1 may again have been primarily occupied during the wet season, and that it served as a temporary hunting campsite. Igneous stone tools from TB1 (Fig 9) are generally green-blue or gray. Most specimens lack visible inclusions, but several appear to have small crystalline inclusions. Specimens for this study include 41 complete flakes, 4 cores, 11 flake fragments, 24 pieces of shatter, 4 utilized cores, 2 angular chunks and 1 complete cobble.

**Fig 10:**
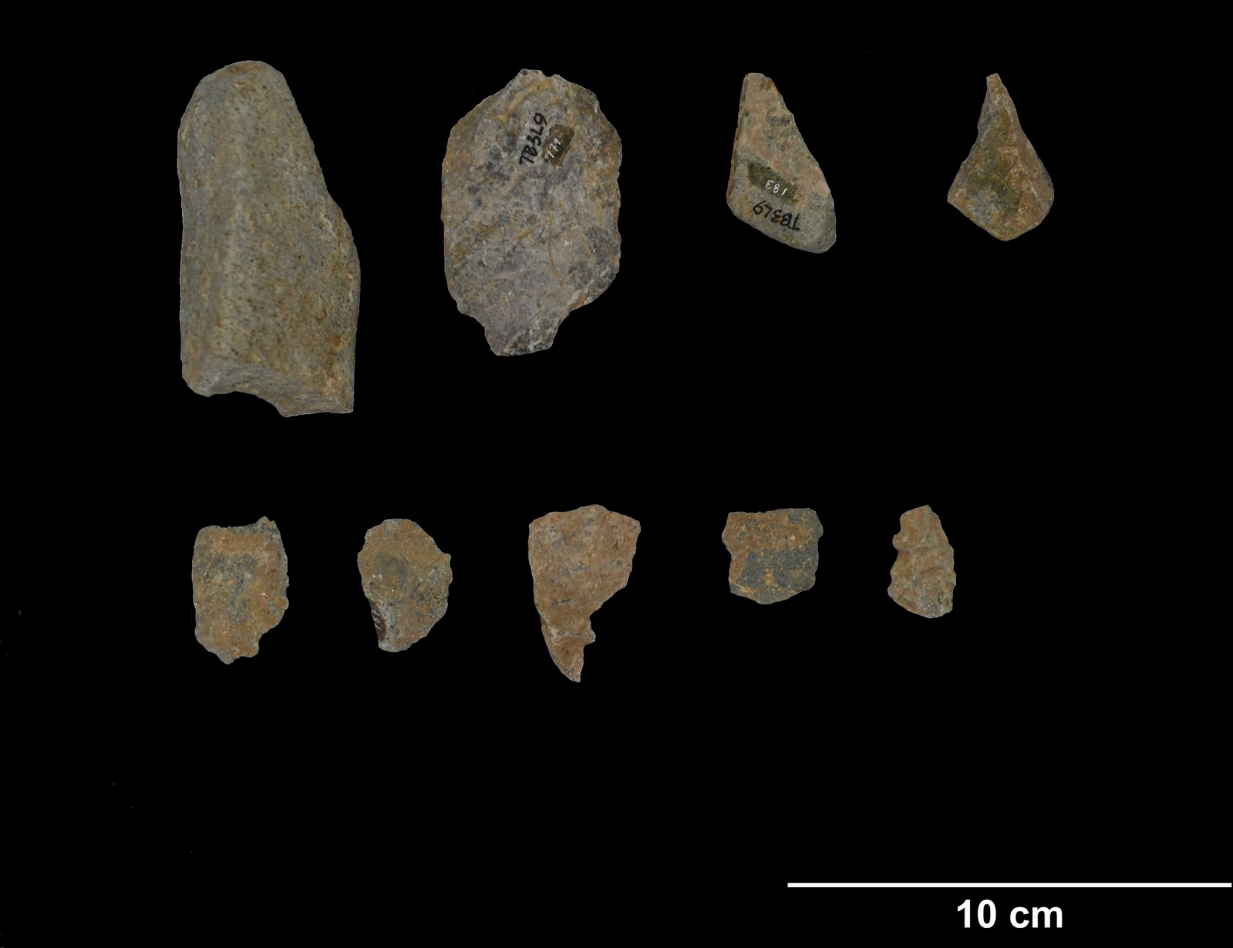
Lithics from Thung Binh 3 (clockwise from upper left): Core tool, flake tools (photo by author).

TB3 (20.26084N, 105.86461E) is situated in the same karstic formation as TB1 and was excavated by the Vietnam Institute of Archaeology in 2012. Radiocarbon dates from TB3 indicate occupation from 15.5–13.5 uncal. BP. [62]. These dates were taken from freshwater *Brotia* sp. shell, for which a calibration curve is currently unavailable. Specimens in this study were excavated from a *Cyclophorus* shell midden rich with other faunal elements, including marine and freshwater mollusks and larger mammalian fauna, notably Cervidae and Bovidae [63]. The zooarchaeological evidence complements that seen at other sites in the Thung Binh massif and appears to be consistent with a hunting camp or butchery site with a likely emphasis on wet season occupation. Igneous stone tools from TB3 (Fig 10) appear to be made of similar green-blue to gray raw material as those from TB1. Specimens for this study include 22 complete flakes, 3 cores, 4 flake fragments, 4 pieces of shatter, 3 utilized cores, and 1 angular chunk.

## Methods

Portable X-Ray Fluorescence Spectroscopy (pXRF) is a technique used in geochemical composition analysis. It is particularly attractive for archaeological applications because it is non-destructive. While other techniques such as thin sectioning or LA-ICP-MS are more accurate, they inflict irreparable damage to specimens [64, 65]. XRF has been successfully used to geochemically fingerprint basalt [66–70], but there are two major considerations to address. The first is that stone tools from Tràng An were fashioned from cobbles almost certainly originating in stream or river deposits. Natural weathering processes can alter elemental concentrations in basalt, with studies indicating that major elements are more effected than trace elements [71]. To mitigate the effects of weathering on analysis, light major elements (e.g. Na, Si, K, Ca) were not included in this investigation. Furthermore, titanium, one of the most diagnostic elements in this study, is known to resist weathering effects extremely well [72–75]. The second major consideration is that readings were recorded from inconsistently shaped surfaces and textures. While an effort was made to use flat, uniform surfaces [76], natural contours in some of the samples meant that, while the x-ray tube and detector were completely covered, some readings were taken from surfaces with irregular surface geometry. The relatively high number of specimens paired with multiple readings recorded from each specimen mitigated these potential sources of inconsistency [77].

Major elements selected for analysis were converted to oxides in weight % to be consistent with published data and included TiO_2_ and Fe_2_O_3_. Trace elements were calibrated as metals in parts per million (ppm) and included Ni, As, Rb, Sr, Y, Zr, Nb, Sn, Sb, Ba, and Th. A table of elemental readings is available in the data supplement (S1 File).

A Bruker Tracer-IV portable XRF machine was used to characterize the elemental composition of the study sample. Each specimen was cleaned with a brush, rinsed with purified water, and dried prior to analysis. Despite these efforts, several readings were rendered inaccurate due to post-depositional residues (e.g., breccia, calcreted soil, other carbonate accretions). These readings were discarded prior to quantitative analysis.

Major elements were characterized by recording three readings from different locations on each specimen for 180 seconds each. XRF machine settings were 15 volts/25 μA (microampere) with no filter inserted. For trace elements, three readings were taken from different locations for 60 seconds each. The XRF machine was set at 40 volts/12.7 μA with a 12 mm Al/1 mm Ti (yellow) filter to reduce background noise. The XRF machine was controlled by Bruker pXRF software, including X-Ray Ops (version 1.2.21) to manage instrument settings and S1PXRF (version 3.8.3.0) for spectrum collection, storage, and export. Raw readings were calibrated using USGS standards (AGV-1 powder, BCR-2 pellet, BCR-2 powder, BHVO-2 powder, G2 powder, GSP-1 powder, and STM-1 powder). Viable readings were then averaged into a single point for each specimen.

### Data analysis

Data analysis sought firstly to categorize specimens based on geochemical composition. Previous archaeological analyses of stone tools from Tràng An have classified igneous stone as rhyolite, basalt, and andesite, but macroscopic identification methods are rarely adequate for classifying raw material. When comparing raw materials for the purpose of geological source discrimination, it is important to compare the same types of raw material (e.g. basalt to basalt or andesite to andesite). The second goal was to identify patterns within each geological subgroup using cluster analysis. The final goal was to assess the hypothesis that igneous archaeological specimens could be attributed to the Cam Thuy formation.

For the first goal, a geochemical discriminant diagram was used to plot diagnostic elemental concentrations against each other to elementally categorize specimens in the study sample into distinct rock types. The most common geochemical discriminant diagram for the classification of igneous stone is the total alkali silica (TAS) diagram, which compares the combined weight of NaO_2_ and K_2_O against the weight of SiO_2_. However, these elements are not reliably measured by pXRF, and are strongly affected by long-term weathering [77]. Instead, the Pearce W-F diagram [78, 79] was used following its’ previous application to archaeological pXRF data [80]. For this study, the primary advantage of the Pearce W-F diagram is that it uses immobile mid atomic number elements that are measured accurately by pXRF (Zr, Ti, Nb, Y).

Principal Component Analysis (PCA) is statistical clustering method used to simultaneously minimize information loss and increase interpretability of multivariate datasets by transforming large sets of variables into smaller components [81]. It has been previously and successfully applied as a method to analyze XRF data of stone tools [66, 67, 82, 83]. Here, it is used to explore variability within raw material subgroups.

For the third goal, boxplots and biplots were constructed to examine variability within geochemical subgroups at more specific resolution, and to compare elemental data from the study sample against known values (e.g. titanium wt%). Boxplots were used to present variability in single elements, and biplots were used to show clustering based on elemental ratios.

Statistical analysis for this study was performed in R [84]. Graphs were constructed with the package ggplot2 [85]. Code and data are available in the supplement (S1 and S2 Files).

## Results

The following sections will present 1) the geochemical classification analysis, 2) cluster analysis including all elemental data, and 3)specific diagnostic elemental data.

### Geochemical classification

**Fig 11:**
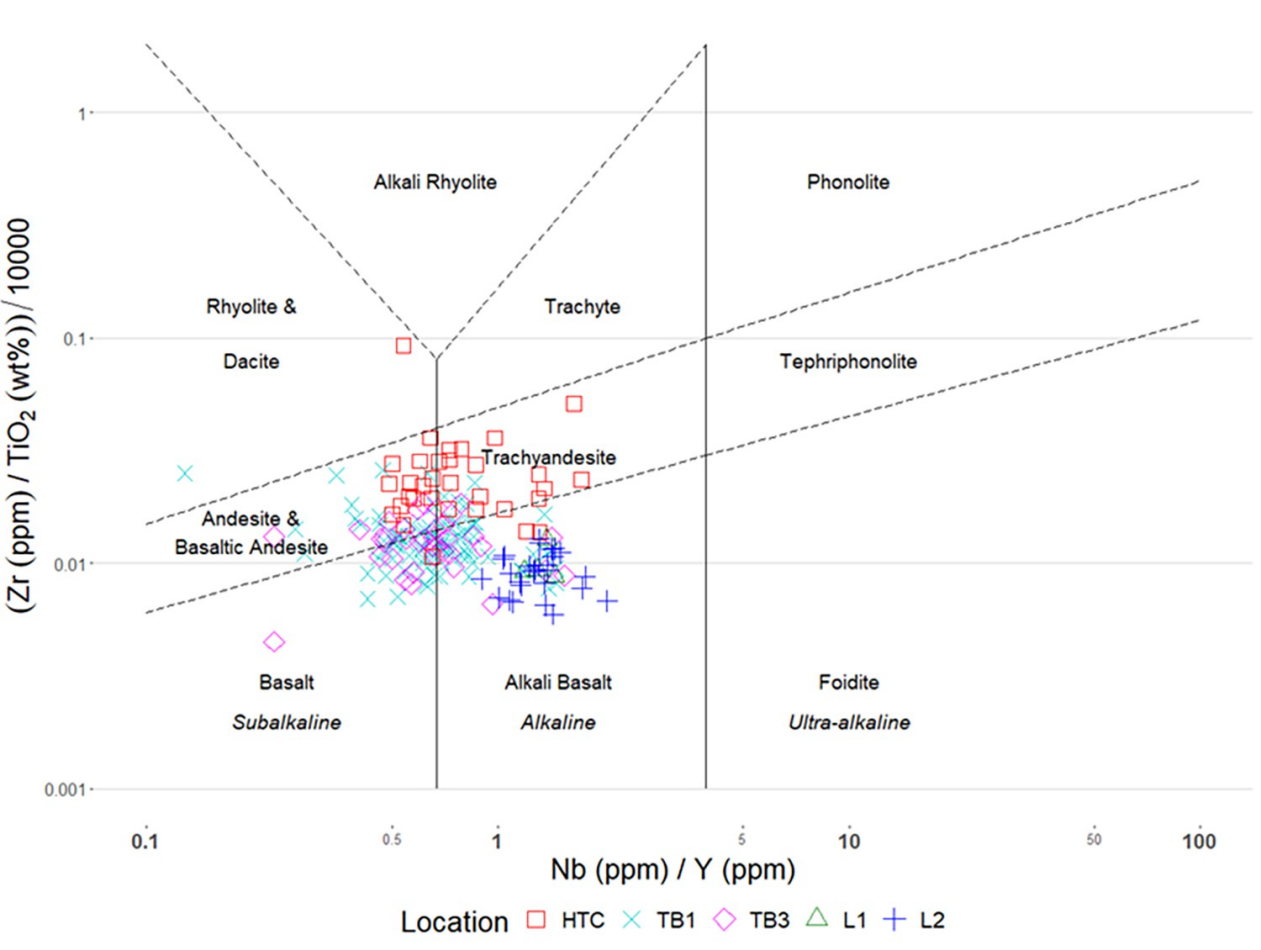
Pearce W-F diagram [78–80].

When plotted on a Pearce diagram (Fig 11), specimens from this study cluster into different areas. Reference samples from Pù Luông clearly cluster into the alkali-basalt group while archaeological samples cluster elsewhere. Archaeological specimens from Hang Trong site have higher Zr/TiO_2_ ratios, placing them into the andesitic category. Specimens from the TB1 and TB3 sites are similar to one another, and largely cluster between the subalkaline basalt and alkaline-basalt categories. A chi-square test with Monte Carlo p-value simulation to adjust for low cell counts [86] indicated statistically significant differences (p < 0.001) in the types of igneous raw material used to make stone tools at different archaeological sites (Table 2).

**Table 2:** Raw material proportions classified by Pearce W-F diagram (percentages in parentheses).

Site	Alkali Basalt	Andesite/Basaltic Andesite	Basalt	Rhyolite	Trachyandesite
HTC (n = 33)	2 (6.1%)	14 (42.4%)	1 (3%)	1 (3%)	15 (45.5%)
TB1 (n = 87)	35 (40.2%)	20 (23%)	23 (26.4%)	1 (1.1%)	8 (9.2%)
TB3 (n = 37)	11 (29.7%)	11 (29.7%)	11 (29.7%)	0	4 (10.8%)
L1 (n = 4)	4 (100%)	0	0	0	0
L2 (n = 27)	27 (100%)	0	0	0	0

### Cluster analysis

**Fig 12:**
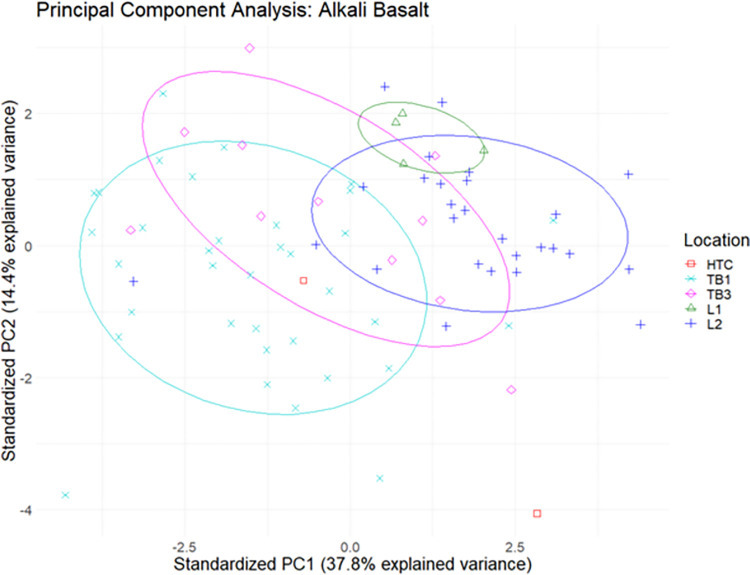
Biplot of principal components 1 and 2 of elemental data with 95% confidence ellipses for alkali basalt.

**Fig 13:**
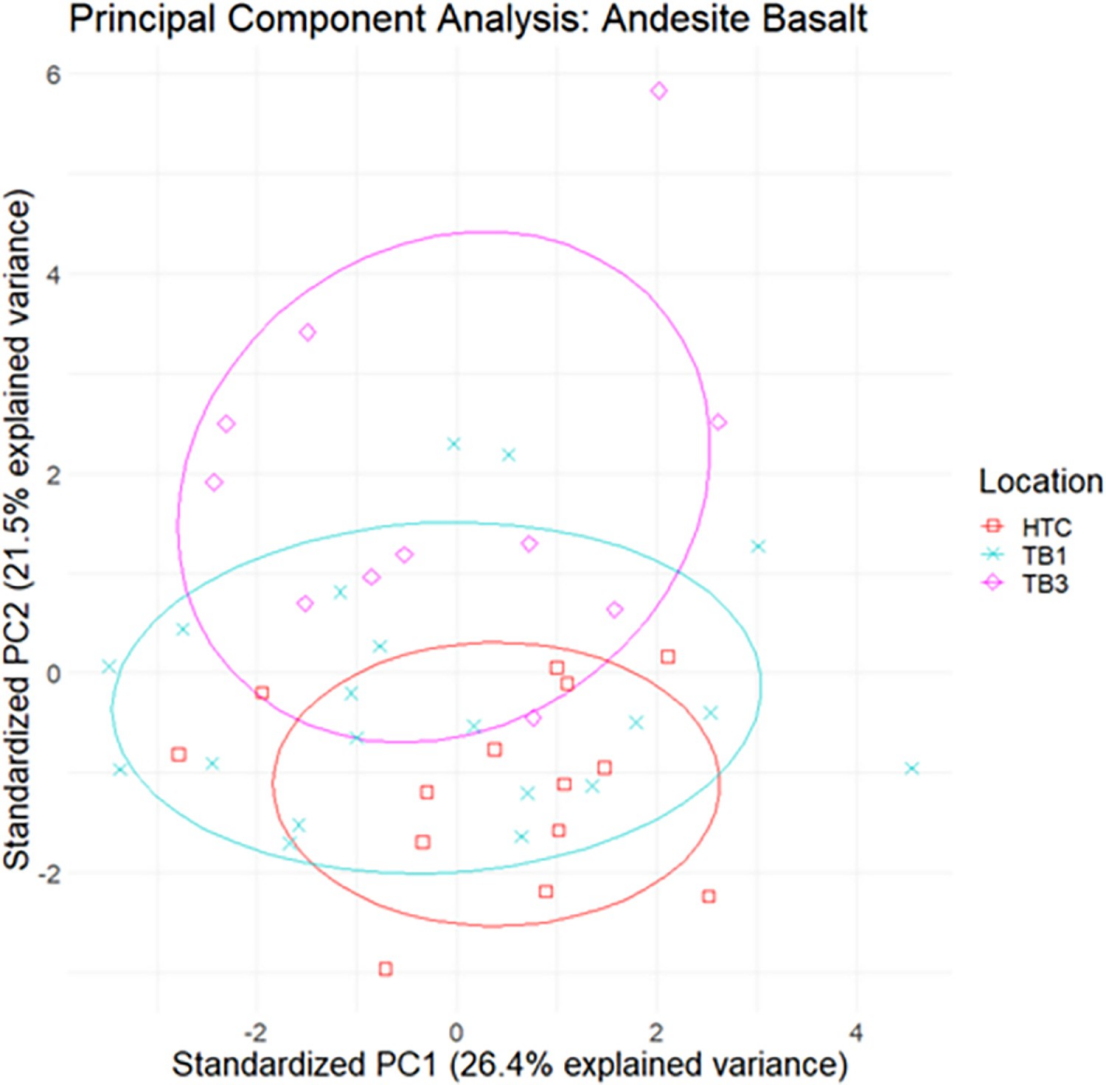
Biplot of principal components 1 and 2 of elemental data with 95% confidence ellipses for andesite basalt.

**Fig 14:**
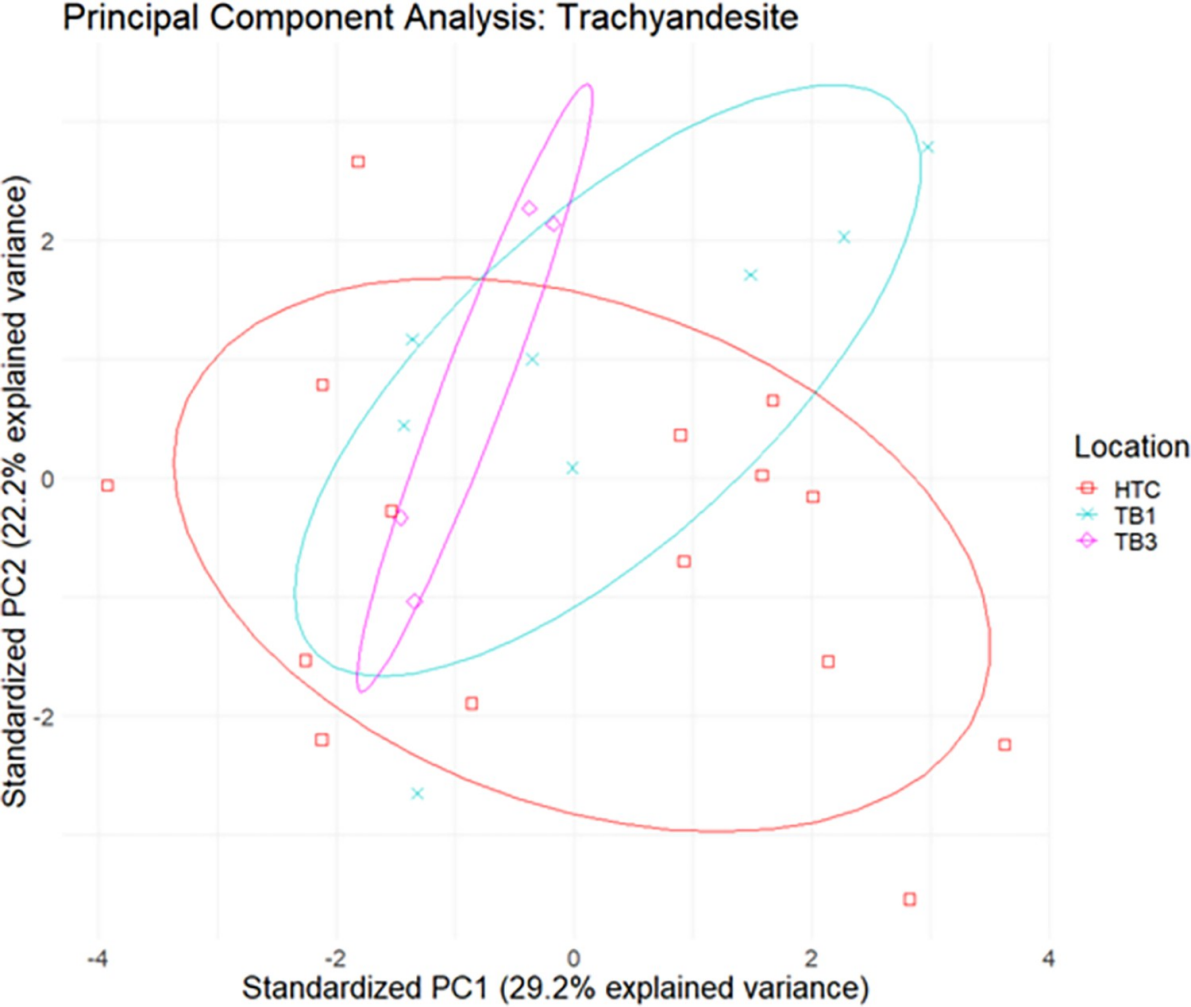
Biplot of principal components 1 and 2 of elemental data with 95% confidence ellipses for trachyandesite.

Study specimens were separated into categories based on the Pearce W-F diagram, and further cluster analysis was conducted on elemental data within the alkali basalt, andesite basalt, and trachyandesite subgroups (Figs 12–14). While there are degrees of overlap within each subgroup, specimens appear to cluster based on geological location and archaeological site. The relatively large distances between datapoints in this series of analyses can likely be attributed to the naturally heterogeneous composition of basalt.

### Elemental concentrations

**Fig 15:**
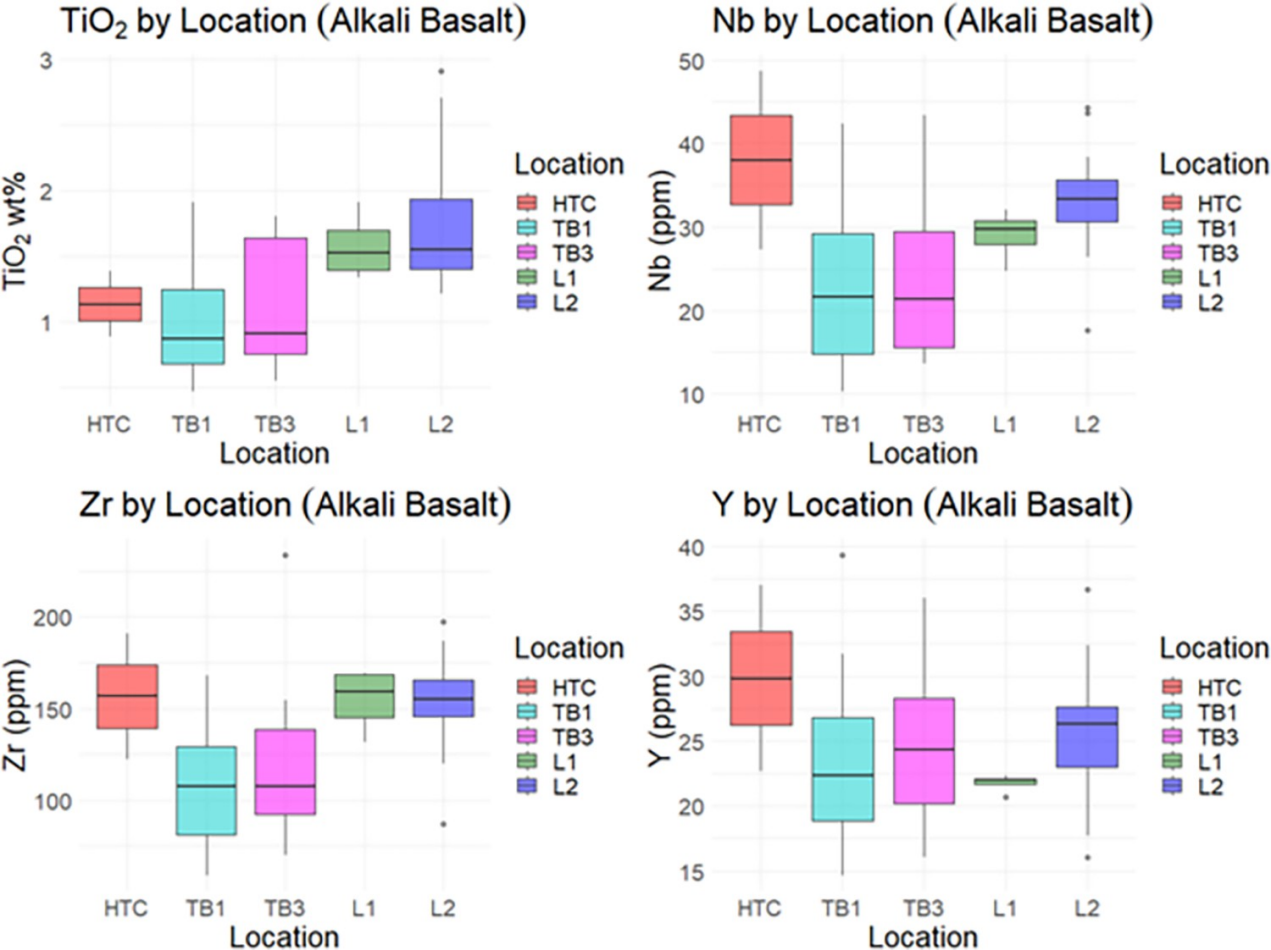
Elemental concentrations by location (alkali basalt).

**Fig 16:**
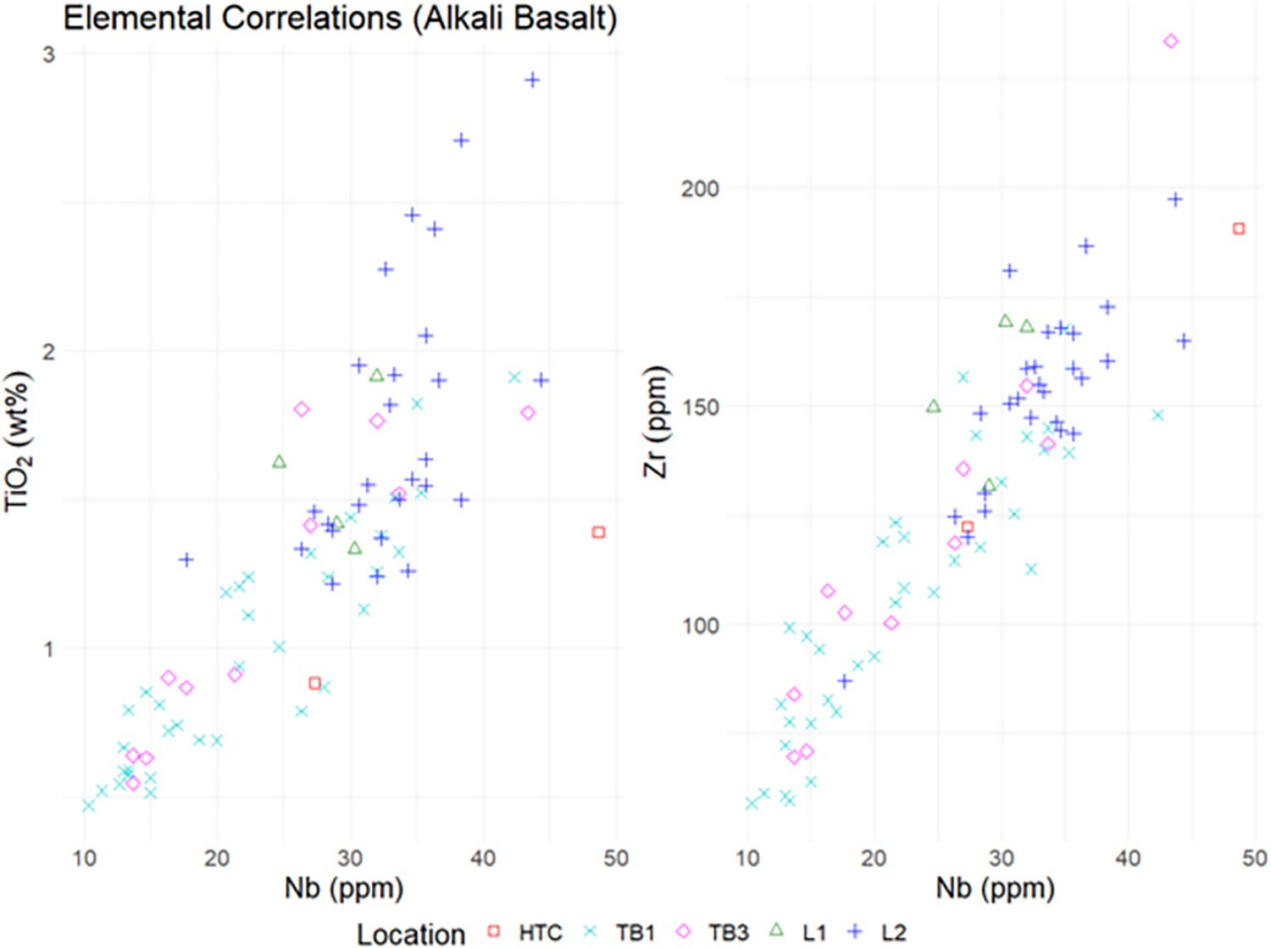
Correlation of Nb (ppm) to TiO_2_ (%wt) and Zr (ppm) to TiO_2_ by location (alkali basalt).

Mean elemental concentration values differed between archaeological assemblages and field-collected geological samples, most notably in TiO_2_, Nb, and Zr (Figs 15 and 16). Geochemical data appear to cluster based on geographical location. Archaeological specimens made of alkali basalt have lower TiO_2_ content than the geochemical samples.

## Discussion

The results clearly show that Pleistocene stone tools from Tràng An were fashioned from a wide variety of igneous raw materials, including rhyolite, alkali basalt, subalkaline basalt, andesite/andesitic basalt, and trachyandesite. Furthermore, comparison of titanium concentrations within the alkali basalt subgroup indicates that the majority of the archaeological samples were *not* made of Cam Thuy basalt, thus partially refuting the hypothesis that igneous stone tools from Tràng An could be geochemically attributed to that formation, as was assumed for the igneous material recovered from nearby Con Moong [59].

Based on comparisons between the archaeological sample and available major elemental data from published literature, it was found that igneous stone tools from Thung Binh 1 and Thung Binh 3 could be attributed to several viable candidate formations, including Song Ma metabasite/gabbro, low-Ti Song Da basalt, and Cam Thuy basalt. Igneous stone tools from Hang Trong were largely geochemically distinct from those from TB1 and TB3 and could be attributed to a combination of Cam Thuy basalt, rhyolite-dacite, and andesite-trachyandesitic stone. The closest source of low-Ti Song Da basalt is near the Hoa Binh hydropower dam, roughly 85 kilometers northwest of Tràng An in Hòa Bình province [53]. However, it is likely that river networks transport low-Ti basalt cobbles and other igneous material towards Tràng An. The geological source(s) of the low-Ti rhyolite, andesite, and trachyandesite remains unresolved.

**Fig 17:**
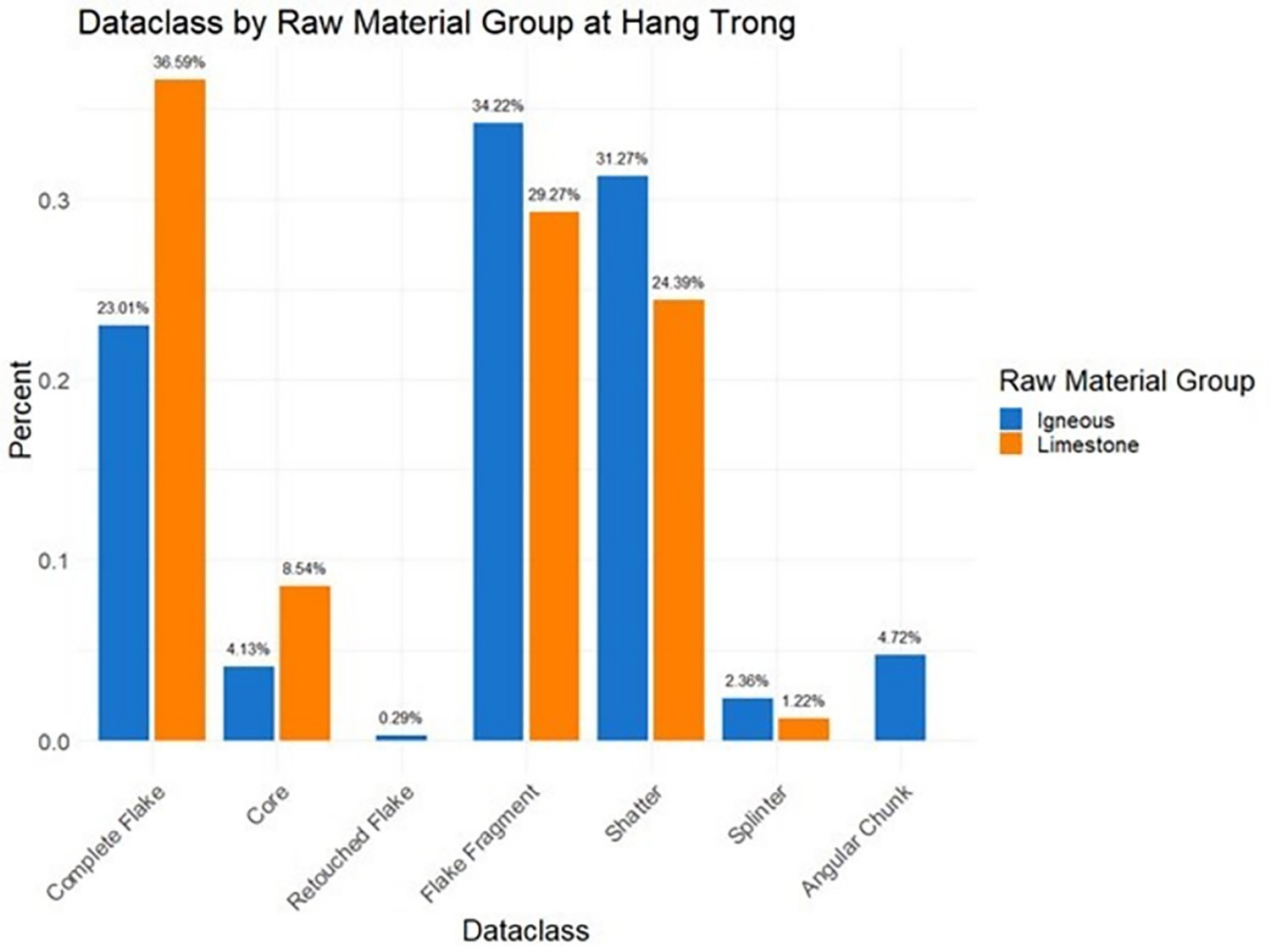
Dataclass by raw material group at Hang Trong, analysis restricted to Pleistocene contexts (n = 421).

**Fig 18:**
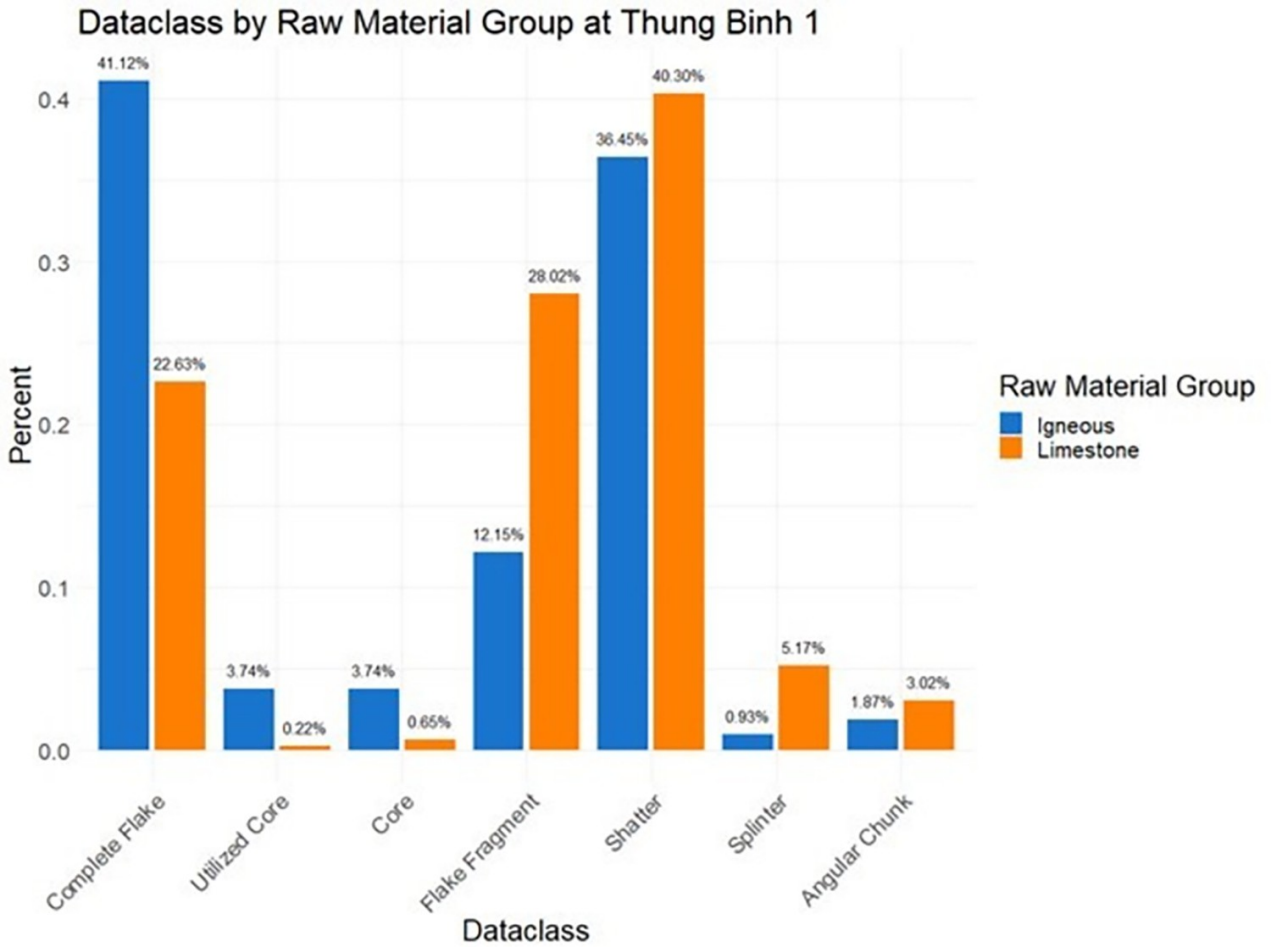
Dataclass by raw material group at Thung Binh 1, analysis restricted to Pleistocene contexts (n = 571).

**Fig 19:**
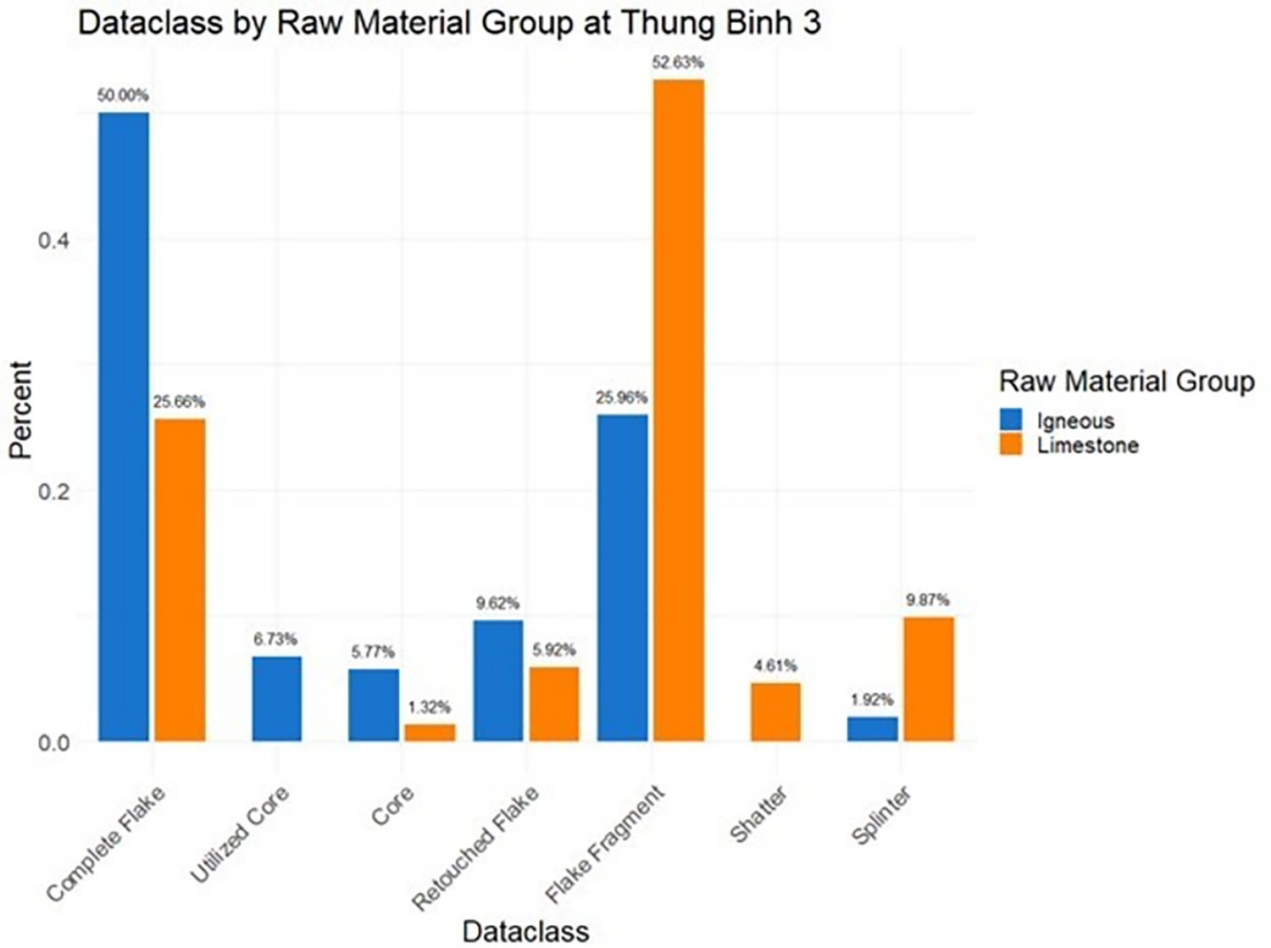
Dataclass by raw material group at Thung Binh 3, analysis restricted to Pleistocene contexts (n = 256).

Comparison of limestone and igneous stone tools (Figs 17–19) helps shed light on technological organization at Hang Trong, Thung Binh 1, and Thung Binh 3. While there are higher numbers of complete flakes and cores in the igneous subsample, both groups include many unretouched, expedient tools and very few retouched tools and utilized cores.

Other lines of archaeological evidence from HTC, TB1, and TB3 are consistent with wet season occupational patterns, though broader seasonal use cannot be ruled out at this time [33, 87]. Zooarchaeological evidence from each site in this study includes a high diversity of faunal resources, biased representation of body parts in some cases (such as distal elements of cervids recovered from HTC [17]), and expediently reduced/lightweight lithic toolkits. There is currently no evidence of storage technologies from this period at sites from Tràng An, but it is likely that any organic storage vessels would not have been preserved. In concert, archaeological evidence (particularly the abundance of cyclophorid shell) is indicative of a primarily wet-season occupation. However, macro-botanic evidence from sites throughout the complex, including Hang Trong, hints at wider seasonal use [88], and it is possible that the more archaeologically visible wet-season linked cyclophorid accumulations overwhelm evidence of a dry-season component to site use. It is also possible that defining mobility based on monolithic divisions of wet and dry season site-use may be missing subtler patterning, such as strategies of mobility that are linked to particular intervals within or close to the boundaries of these dominant seasonal types and linked to resource availability and accessibility.

The presence of non-local raw materials (and the recovery of a whole cobble from TB1) is potentially indicative of self-provisioning of raw material, though the relationship between non-local raw material and seasonal mobility patterns remains for the moment unclear. It is possible, for example, that non-local raw materials are indicative of logistic foraging expeditions to areas where igneous raw materials are available, or of large-scale residential mobility strategies during the wet season. As noted, subtler seasonally related mobility may also factor. Reasons for the investment involved in the transport of igneous cobbles and/or tools around the landscape remain unknown. However, it is possible that the use of rafts or small boats [89] significantly reduced any energetic costs associated with the transportation of raw material, and that whole cobbles or tools could be easily transported throughout the dense riverine network of northern Vietnam, either as an aspect of group mobility or exchange mechanisms.

It is also important to note that the observed technological differences between assemblages are not necessarily driven by deliberate behavioral decisions in the reduction process. Limestone at Tràng An is relatively hard and suitable for stone tool manufacture but frequently has substructural inclusions and is prone to fracturing unpredictably. Therefore, otherwise identical stone tool reduction methods might result in different observed outcomes for limestone and igneous cobbles. Further experimental research into the knapping characteristics of limestone and basalt, matched with the analysis of zooarchaeological and botanical remains will be necessary to help further explain the archaeological record in Tràng An and the impact that seasonality had more generally on the kinds of tropical mobility strategies hinted at in this study.

There is a considerable degree of elemental variability between igneous specimens from Hang Trong and the Thung Binh caves, which is also reflected in macroscopic differences in color and texture. The former is older than both TB1 and TB3, and the results of this study might indicate changes in the circulation of stone, by whatever means, before and after the Last Glacial Maximum (26–19,000 years ago) [45, 46]. However, technological analysis suggests similar reduction patterns of raw materials between the Thung Binh caves and Hang Trong. Thus, while sources of igneous cobbles appear to have changed after the Last Glacial Maximum, technological approaches to reducing stone remained largely the same. Further analysis of archaeological material should focus on diachronic and synchronic raw material variability at sites throughout the complex.

Portable XRF has limited analytical resolution, and some lighter diagnostic elements that might further narrow down the geological source (e.g., sodium, potassium, silicon, magnesium) are not detected well by this method [90]. The application of more precise methods, including LA-ICP-MS [83] and thin sectioning [59, 91], might help shed more light on the geographic source of the archaeological igneous material, though will carry the downside of being destructive.

## Conclusion

Historical typological approaches defined mainland Southeast Asian stone tool assemblages by a series of diagnostic retouched core types. However, a growing body of evidence suggests that the small, expedient, and unretouched elements that dominate these assemblages are at least as important as the larger, retouched elements. Therefore, research into the lesser studied components of these assemblages has the potential to contribute significantly to a more comprehensive understanding of behavioral variability in prehistoric mainland Southeast Asia.

This study has revealed that, despite the ubiquity of locally available technologically suitable raw material, the prehistoric inhabitants of Tràng An invested in obtaining igneous stone from a relatively long distance: perhaps as far as 85 km away. Despite this investment, they seemingly did not reduce this non-local stone in any significantly different manner than from local limestone. The reasoning behind this series of technological choices is unknown, though this study provides clear evidence that a notable level of behavioral complexity underlies the apparent simplicity of these tool kits. Further application of geochemical techniques will help identify more precisely the geographic locations of exploited igneous outcrops (and if these changed through time) and help to build a more detailed picture of forager mobility and technological decision-making that will have wider relevance for our understanding of Palaeolithic societies in this part of the world.

## Supporting information

S1 File Raw data for reproducing analysis with specimen numbers. (CSV)

S2 File R Code for reproducing statistical analyses and graphs. (R)
